# Multimodal treatment for smoking cessation with varenicline in alcoholic, methadone-maintained, and psychotic patients: A one-year follow-up

**DOI:** 10.18332/tid/99541

**Published:** 2018-12-11

**Authors:** Antonia Raich, Cristina Pinet, Montse Ballbè, Silvia Mondon, Rosa Tejedor, Anna Arnau, Esteve Fernández, on behalf of the Tobacco Group of the Catalan Network for Addiction Treatment

**Affiliations:** 1 Group of Research SAMIS, Mental Health Department, Althaia Xarxa Assistencial Universitària de Manresa, Barcelona, Spain; 2 Department of Clinical Sciences, School of Medicine, Universitat de Barcelona, Barcelona, Spain; 3 Addiction Unit, Mental Health Department, Hospital de Sant Pau, Barcelona, Spain; 4 Addictions Unit, Psychiatry Department, Neurosciences Institute, Hospital Clínic, IDIBAPS, Barcelona, Spain; 5 Tobacco Control Unit, Institut Català d’Oncologia, IDIBELL, Barcelona, Spain; 6 Research Department, Althaia Xarxa Assistencial Universitària de Manresa, Barcelona, Spain

**Keywords:** varenicline, multimodal treatment, psychotic, addicted patients

## Abstract

**INTRODUCTION:**

Numerous studies have evaluated the efficacy and safety of varenicline for smoking cessation in smokers in the general population and, to a lesser extent, among the psychiatric population. However, few studies have evaluated varenicline in patients with other addictions. The present study was conducted to assess outcomes of a multimodal treatment for smoking cessation intervention with varenicline in a sample of alcohol and substance use disorders and patients with psychotic disorders.

**METHODS:**

This was a prospective, multicenter study. The patient sample comprised alcoholics in remission, methadone-maintained patients, and patients with psychotic disorders, all of whom wanted to stop smoking. All participants received multimodal treatment for smoking cessation therapy (psychological therapy plus varenicline). Smoking abstinence and changes in the psychopathological state of patients were assessed at predefined time points during a 12-month follow-up. The probability of tobacco abstinence after one year of treatment was computed using Kaplan-Meier life tables.

**RESULTS:**

The probability of abstinence at one year was 0.225 (95% CI: 0.1430-0.319). By group, the probabilities were as follows: patients with psychotic disorders 0.254 (95% CI: 0.118-0.415); alcoholics 0.237 (95% CI: 0.098-0.409); and methadone-maintained patients 0.177 (95% CI: 0.065-0.335). Patients with previous quit attempts had a higher probability of achieving abstinence at one year (p<0.01).

**CONCLUSIONS:**

The results of this study support the use of multimodal treatment with varenicline in patients with alcohol addiction in remission, patients on methadone maintenance, and patients with stable psychotic disorders. Previous smoking cessation attempts were predictive of smoking cessation success in these patients.

## INTRODUCTION

Tobacco use is a risk factor in six of the eight leading causes of death in the world (World Health Organization, 2003). Worldwide, tobacco use kills one person every 6 seconds and reduces life expectancy by an average of 15 years^1^. Despite the significant decline in smoking prevalence in the general population in recent decades, the prevalence among psychiatric patients remains high. In a study with a North American population, the prevalence among patients who had ever had mental illness is high (55% of lifetime smokers and 35% of current smokers^2^. The highest smoking rates are seen in individuals diagnosed with schizophrenia, mania, or depression^3^. Moreover, studies have shown that there is a direct relationship between the intensity of the psychiatric condition and the severity of the smoking habit^4^. Estimates suggest that 43-80% of alcohol abusers and 49-98% of individuals with substance use disorders smoke cigarettes^5^.

Tobacco smoking causes considerable morbidity and mortality in patients with severe mental illness, with published reports suggesting that the high mortality rates associated with schizophrenia can largely be attributed to smoking^6^. Compared to the general population, people with severe mental illness are two to three times more likely to suffer from smoking-related illnesses such as cardiovascular disease or cancer^7^.

Approved Food and Drug Administration (FDA) first-line medications for treating tobacco use include nicotine replacement therapies, bupropion SR, and varenicline^8^. However, these treatments are more effective when combined with psychological treatment. Systematic reviews confirm that treatment interventions based on behavioral support combined with pharmacotherapy are effective in the general population and in smokers with mental illness and addictive disorders^9,10^; importantly, these interventions do not appear to worsen psychiatric symptoms^11^.

Varenicline, an alpha-4-beta-2 nicotinic acetylcholine receptor (nAChR) partial agonist^12^, was first commercialized in 2006 and has been approved by both the FDA and the European Medicines Agency (EMA). This receptor has been linked to the reinforcing effects of nicotine and to continued smoking behavior. Varenicline appears to partially reproduce the action of nicotine, with a dual mechanism of action, as it is a partial agonist with higher affinity but less functional effect than nicotine. As a result, varenicline may alleviate both craving and withdrawal symptoms during smoking cessation. In addition, if nicotine exposure occurs, varenicline would be expected to block the reinforcing effects of smoking by binding to the same receptor^13^.

Several studies have assessed the efficacy and safety of varenicline and its relative effectiveness versus other drugs for smoking cessation. Varenicline is significantly more effective than placebo and more efficacious than bupropion and nicotine replacement therapy (NRT)^14,15^. The safety and efficacy of varenicline have been well-demonstrated in smokers in the general population and in those with smoking-related pathologies (e.g. cardiovascular disease and lung diseases)^16,17^. However, the benefits of varenicline in smokers with psychotic disorders were only recently confirmed with the publication of the randomized clinical trial conducted by Anthenelli et al.^11^, known as the EAGLES trial. Most studies conducted to date have involved patients without mental illness or addictions. In fact, the presence of these disorders is often considered exclusion criteria in clinical trials. In the EAGLES trial, at 24 weeks follow-up, varenicline was more efficacious in achieving smoking cessation than nicotine patches, bupropion, or placebo. Notably, that study included patients with psychiatric disorders but excluded patients with drug use disorders within the previous 12 months. Therefore, the efficacy of varenicline in patients with substance use disorders—who have a high prevalence of smoking and severe difficulty in quitting—remains unknown. A population-based study compared varenicline to nicotine replacement therapy and concluded that varenicline does not seem to be associated with a higher risk of depression or self-harm; however, that study used data from patients in the validated Q Research database and excluded patients who used drugs during the 12-month period prior to initiation of the study^18^.

The process of quitting smoking, with or without medical treatment, has been associated with the emergence of psychiatric symptoms such as depression, dysphoria and anxiety, all of which are part of the nicotine withdrawal syndrome^19^. Importantly, smoking itself—particularly heavy smoking—has been closely linked to suicidal ideation and suicidal behavior in several studies^20^.

Given the paucity of real-world data on the safety and efficacy of varenicline among psychotic patients, alcoholics, and patients with substance use disorders, we examined the outcomes of a smoking cessation program involving multimodal intervention (varenicline plus psychological treatment) after one year of follow-up. The sample included alcoholic patients in remission, patients on methadone-maintenance programs, and patients with psychotic disorders. We also sought to determine if this multimodal treatment was associated with changes in the psychiatric or addictive disorders of these patients.

## METHODS

### Study design and subjects

This was a prospective, longitudinal study carried out at 11 specialized tobacco addiction units within the substance abuse treatment network of Catalonia, Spain. Participants were recruited from patients being treated at these centers who expressed a desire to quit smoking. Recruitment took place between September 2008 and December 2009. Patients were included consecutively following their request to participate in the study.

A total of 90 patients with a confirmed diagnosis of nicotine dependence were recruited. All participants had a diagnosis of one of the following disorders: alcohol dependence disorder, opioid dependence disorder on methadone-maintenance, or psychotic disorder (schizophrenia, schizoaffective disorder, or chronic delusion).

Inclusion criteria were: 1) ≥ age 18 years; 2) expressed a desire to quit smoking; 3) preserved cognitive abilities that allow to follow psychological therapy; 4) clinically stable (free of acute decompensation during the past 6 months); 5) stable pharmacological treatment for ≥ one month; and 6) active smoking habit of ≥ 10 cigarettes/day during the 12 month prior to enrolment in the study. Exclusion criteria were: 1) diagnosis of other abuse or dependence disorders in the last 6 months; 2) clinically significant medical disease in the preceding 6 months for which varenicline is contra-indicated; 3) previous history of suicidal behavior or presence of suicidal risk at screening; 4) diagnosis of other DSM IV axis I psychiatric disorders; and 5) pregnancy.

Based on standard calculations, the required sample size was estimated to be 129 patients. However, only 90 were included in our study, due to the limited budget assigned for this purpose by the Department of Health of the Government of Catalonia, which was funding the treatment.

The local ethics committees approved the study protocol. Patients were informed about the study protocol and provided written informed consent to participate in the study.

### Measurements

Sociodemographic data were collected during the baseline interview. The following smoking characteristics were assessed: number of cigarettes smoked per day in the last month, years of smoking habit, and number of previous attempts to quit. We also registered all psychiatric diagnoses and current pharmacological treatments. Exhaled carbon monoxide (CO) was measured with the piCO+ Smokerlyzer^®^ (Bedfont Scientific, Kent, England). Nicotine dependence was assessed with the Spanish version of the Fagerström Test for Nicotine Dependence (FTND). We gathered the patients’ level of motivation and confidence in their ability to quit smoking. The therapist asked them to score it in a visual analogue scale (VAS) ranging from 0 to 10. To measure the motivation, they were asked: ‘From 0–10 mark the level of importance given by you to quitting smoking’, and to measure self-confidence: ‘From 0-10 mark how confident you are you will quit smoking in the next months’. All variables described above were categorized for the data analysis (Table 1).

**Table 1:** Patient baseline sociodemographic and smoking characteristics

	*Full cohort (n=90)*	*Alcohol disorder (n=29)*	*Methadone maintenance (n=30)*	*Psychotic disorder (n=31)*	*p*
**Demographic characteristics**					
**Sex**, n (%)					
Male	64 (71.1%)	18 (62.1%)	22 (73.3%)	24 (77.4%)	0.401
Female	26 (28.9%)	11 (37.9%)	8 (26.7%)	7 (22.6%)	
**Age (years)** mean (SD)	44.8 (10.1)	50.7 (10.5)	42 (7.3)	42 (9.8)	0.000
**Physical illness**, n (%)					0.031
No concomitant diseases	38 (46.3%)	11 (40.7%)	16 (59.3%)	11 (39.3%)	
Cardiovascular disease	8 (9.8%)	6 (22.2%)	1 (3.7%)	1 (3.6%)	
Respiratory disease	14 (17.1%)	5 (18.5%)	3 (11.1%)	6 (21.4%)	
Cancer	2 (2.4%)	0	1 (3.7%)	1 (3.6%)	
Infectious diseases	7 (8.5%)	1 (3.7%)	5 (18.5%)	1 (3.6%)	
Others	13 (14.4%)	4 (14.8%)	1 (3.7%)	8 (28.6%)	
**Smoking characteristics**					
**Duration of smoking (years)** n (%)					0.005
<20	24 (27%)	3 (10.3%)	8 (26.7%)	13 (43.3%)	
20−30	32 (36%)	8 (27.6%)	14 (46.7%)	10 (33.3%)	
>30	33 (37.1)	18 (62.1%)	8 (26.7%)	7 (23.3%)	
**Cigarettes per day (last month)** n (%)					0.026
<20	13 (14.4%)	6 (20.7%)	6 (20.7%)	1 (3.2%)	
20−30	53 (58.9%)	17 (58.6%)	20 (66.7%)	16 (51.6%)	
>30	24 (26.7%)	6 (20.7%)	4 (13.3%)	14 (45.2%)	
**Cigarettes per day**, mean (SD)	26.9 (10.8)	25.9 (10.8)	23.1 (9)	31.5 (11)	0.007
FTND* score n (%)					0.237
Low (≤4)	12 (13.5%)	2 (7.1%)	5 (16.7%)	5 (16.1%)	
Medium (5–6)	25 (28.1%)	6 (21.4%)	12 (40.0%)	7 (22.6%)	
High (≥7)	52 (58.4%)	20 (71.4%)	13 (43.3%)	19 (61.3%)	
FTND* score, mean (SD)	6.9 (2.3)	7.2 (2)	6.2 (2.2)	7.2 (2.4)	0.237
**Previous quit attempts**, n (%)					0.272
0	32 (35.6%)	9 (31%)	15 (50%)	8 (25.8%)	
1−2	41 (45.6%)	14 (48.3%)	12 (40%)	15 (48.4%)	
>3	17 (18.9%)	6 (20.7%)	3 (10%)	8 (25.8%)	
**Previous quit attempts**, mean (SD)	1.3 (1.3)	1.4 (1.3)	0.8 (1)	1.7 (1.5)	0.034
**Importance to quit score**, n (%)					0.669
Low (≤5)	1 (1.1%)	0	0	1 (3.2%)	
Medium (6−7)	5 (5.6%)	2 (6.9%)	2 (6.7%)	1 (3.2%)	
High (≥8)	84 (93.3%)	27 (93.1%)	28 (93.3%)	29 (93.5%)	
**Importance to quit score**, mean (SD)	8.6 (1.2)	8.6 (1.1)	8.6 (1.1)	8.6 (1.4)	0.669
**Confidence to quit score**, n (%)					0.042
Low (≤5)	21 (23.3%)	9 (31%)	5 (16.7%)	7 (22.6%)	
Medium (6−7)	29 (32.2%)	3 (10.3%)	14 (46.7%)	12 (38.7%)	
High (≥8)	40 (44.4%)	17 (58.6%)	11 (36.7%)	12 (38.7%)	
**Confidence to quit score**, mean (SD)	6.1 (2.5)	5.9 (3.4)	6.4 (1.9)	6 (2.1)	0.042

* FTND: Fagerström Test for Nicotine Dependence

Abstinence, the main outcome variable, was measured at weeks 1, 2, 4, 8, 12, 26, 38 and 52 after ‘quit day’ by self-reported smoking status and exhaled CO levels. Participants were considered abstinent when they reported not smoking any cigarettes in the last 7 days and when this was confirmed by a CO concentration <6 ppm^21^. At the end of the study in week 52, all were contacted to perform the CO measurement. Those who said they were abstinent made the measurement; only some of those who said they were smoking did not attend the visit at session 8.

At each follow-up visit, the therapist evaluated safety. Adverse events were monitored according to FDA Guidelines and classified on a scale ranging from 0-3; where 0=none, 1=mild, 2=moderate and 3=intense. Changes in the psychopathological state of patients were assessed. Modifications in pharmacological treatment for psychiatric pathologies and adherence to pharmacological treatment to quit smoking were monitored and recorded. The results regarding safety have been previously reported in detail^22^.

### Interventions

The intervention consisted of a 12-month multimodal individual treatment for smoking cessation, a standardized treatment approach that combines both pharmacological and psychological interventions^23^. In all cases, clinical psychologists administered the intervention or by psychiatrists specialized in tobacco addiction. All the therapists were members of the Tobacco and Mental Health working group of the Catalan Network of Smoke-Free Hospitals and had participated in its clinical intervention Guide on the use of tobacco in patients with mental disorders, of which they were co-authors. The protocol applied for the psychological intervention was based on the Guide^24^, its content in line with the available scientific evidence. The Guide was authored by clinicians from almost all Catalan hospitals with mental health wards and was supported by the Department of Health of the Catalan Government.

All participants were scheduled for an initial visit to collect baseline clinical data, to discuss motivational strategies for smoking cessation, and to prescribe pharmacological treatment with varenicline, which began the day after the initial visit. Patients were expected to quit smoking within the 8th to 14th day after starting pharmacological treatment. This day was termed the ‘quit day’ or ‘D-day’. Pharmacological treatment with varenicline was administered for 12 weeks, starting with an initial dose of 0.5 mg per day for 3 days, 0.5 mg twice a day from day 4 to 7, and 1 mg twice a day for the following 11 weeks.

In the next sessions, the therapist continued to work on the motivation for quitting and on relapse prevention strategies. The visit with the psychological intervention plus the monitoring lasted about 45 minutes. Specifically, the session was carried out in the following way: the patients completed a questionnaire before the visit where information was collected on tobacco consumption, pharmacological treatment, adverse effects, changes in psychiatric treatments and all the measures already described. Of the 45 minutes of the session, between 5 and 10 minutes were devoted to reviewing these data and confirming abstinence by measuring CO, the rest were dedicated to motivation and prevention of relapse, according to the above-mentioned Guide.

The entire treatment regimen (pharmacological treatment and the psychological intervention) was delivered free to the patients. Varenicline was provided by the Department of Health (Public Health Directorate) of the government of Catalonia. The multimodal treatment was performed in public health centers of the Catalan network for mental health and addiction treatment.

### Statistical analysis

We used the Kaplan–Meier method to estimate cumulative abstinence (probability of continued abstinence and 95% CI) at one year. We used Cox regression models to estimate the hazard ratios with 95% CI for relapse at the end of the follow-up, after checking proportionality during follow-up. We used the Stata software program, v.14 (StataCorp; College Station, Texas; USA) for data analysis.

## RESULTS

### Sample characteristics

Of the 90 patients included in the sample, 29 presented a disorder for alcohol dependence, 30 were patients addicted to heroin who followed a program of methadone maintenance, and 31 presented a psychotic disorder.

Most of the patients (71.1%) in the three groups were males. The mean age of the patients was 44.8 years. Among the three groups, the mean age of the alcoholic group (50.7 years) was significantly greater than the other two groups. There were no significant differences among the groups in terms of educational level: 22.3% did not complete primary school while 43.3% had a high school diploma. In the methadone-maintenance group, 50% of participants were employed versus 16.1% in the psychotic disorder group (64.5% of which received government assistance for their inability to work).

Overall, 53.7% of the patients had some type of physical illness, the most common being respiratory disease (17.1%), cardiovascular disease (9.8%), and infectious disease (8.5%). In the methadone-maintenance group, 59.3% of the patients had no physical illness, a significantly lower proportion than in the other groups (p=0.031).

The mean number of cigarettes smoked per day in the overall sample was 26.9, with the highest number (31.5) observed in the psychotic disorder group. All of the patients were highly dependent on nicotine (mean FTND=6.9). The mean level of exhaled CO was 25.3 ppm. Most of the sample (64.5%) had made at least one serious previous attempt to quit smoking, although the patients in the methadone-maintenance group made significantly fewer quit attempts than patients in the psychotic disorder group (p=0.034).

Overall, the sample had a mean motivation to quit score on the VAS (0 to 10) of 8.6 points, with a mean score of 6.1 regarding confidence in their ability to successfully quit. The highest confidence levels were observed in methadone-maintenance patients and the lowest in the alcoholic group (p=0.042), as shown in Table 1.

### Quitting smoking

**Figure 1:**
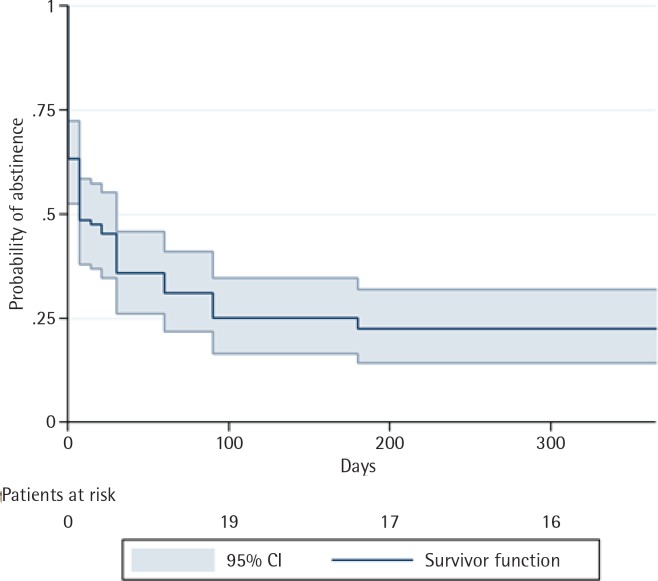
Kaplan–Meier survival curves showing probability of abstinence at one-year of follow-up for all patients

At 52 weeks after the quit day, the probability of quitting smoking was 0.225 (95% CI: 0.143–0.319). The probability of quitting smoking at 1, 4 and 8 weeks after the quit day, whilst still using varenicline, was 0.486 (95% CI: 0.379–0.584), 0.359 (95% CI: 0.261–0.458) and 0.311 (95% CI: 0.219–0.410), respectively. Patients received varenicline for 12 weeks after the quit day. The probability of abstinence at 12 and 26 weeks after quitting were, respectively, 0.251 (95% CI: 0.166–0.346) and 0.225 (95% CI: 0.143–0.318). Notably, this probability at week 26 was the same as that observed at week 52 (Figure 1).

No relapses in psychiatric illnesses were observed in any of the patients who stopped smoking.

The probability of successfully quitting smoking increased as a function of the previous attempts to quit, patients with more previous attempts had higher probability successfully quitting smoking (p<0.01), as shown in Table 2.

**Table 2:** Probability of abstinence and hazard ratios for relapse at one-year follow-up

*Variables*	*Probability (95% CI)*	*p*	*Hazard ratio (95% CI)*
Total	0.225 (0.143−0.319)		
**Diagnostic group**		0.470	
Psychotic disorder	0.254 (0.118−0.415)		1
Alcohol disorder	0.237 (0.098−0.409)		0.87 (0.48−1.58)
Methadone-maintenance	0.177 (0.065−0.335)		1.19 (0.67−2.12)
**Sex**		0.241	
Male	0.250 (0.150−0.363)		1
Female	0.164 (0.052−0.332)		1.3 (0.78−2.18)
**Smoking characteristics**			
**Duration of smoking (years)**		0.786	
<20	0.208 (0.076−0.385)		1
20−30	0.237 (0.107−0.396)		0.83 (0.45−1.52)
>30	0.203 (0.083−0.360)		0.89 (0.49−1.61)
**Cigarettes smoked per day last month**		0.51	
<20	0.192 (0.033−0.450)		1
20−30	0.244 (0.136−0.450)		1.11 (0.56−2.24)
>30	0.194 (0.065−0.376)		1.26 (0.58−2.72)
FTND* score		0.158	
≤4	0.083 (0.005−0.311)		1
5−6	0.338 (0.161−0.523)		0.56 (0.258−1.211)
≥7	0.184 (0.088−0.307)		0.85 (0.435−1.651)
**Previous quit attempts**		0.0009	
0	0.044 (0.003−0.183)		1
1−2	0.208 (0.098−0.347)		0.66 (0.39−1.10)
3−5	0.529 (0.276−0.730)		0.28 (0.12−0.63)
**Importance to quit score**		0.395	
<5	0		1
5−7	0.300(0.123−0.719)		0.30 (0.31−2.94)
>7	0.225(0.141−0.321)		0.36 (0.04−2.66)
**Confidence to quit score**		0.140	
<5	0.159 (0.040−0.348)		1
5−7	0.197 (0.075−0.360)		0.78 (0.41−1.46)
>7	0.277 (0.146−0.423)		0.60 (0.32−1.10)
**Adherence to multimodal therapy**		0.0000	
<4 sessions	0		1
4−7 sessions	0.150 (0.037−0.334)		0.36 (0.19−0.70)
>7 sessions	0.417 (0.262−0.565)		0.15 (0.82−0.30)

* FTND: Fagerström Test for Nicotine Dependence, CI: confidence interval.

The same occurred with multimodal treatment adherence, the patients who came at more sessions had a higher probability to quit smoking (p<0.01), shown in Table 2).

### Adherence

Only 30 of 90 (33.3%) patients attended the 8 sessions, 21 of which were abstinent at one year of follow-up. One patient of the 22 who were abstinent in week 52 attended seven sessions. The remaining nine patients relapsed before the 52 weeks of follow-up. No differences in adherence were observed between diagnostic groups.

### Group differences in the results

**Figure 2:**
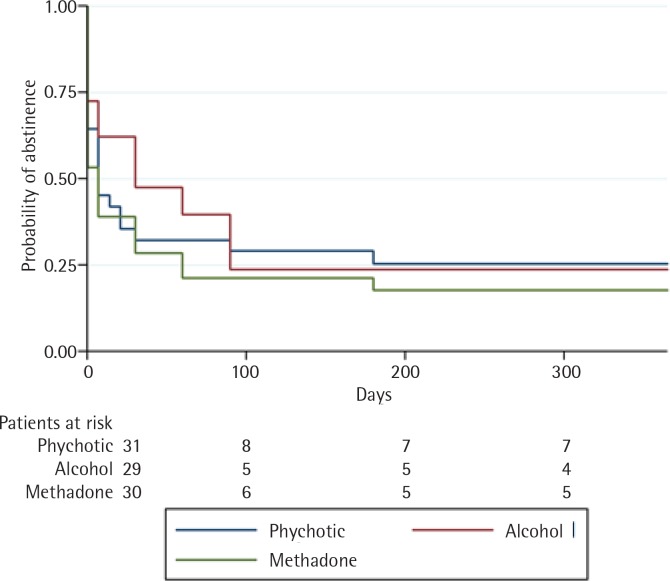
Kaplan–Meier survival curves showing probability of abstinence at one-year of follow-up according to diagnosis

At 52 weeks of follow-up, the highest rate of successful smoking cessation was observed in the psychotic disorder group, at 0.254 (95% CI: 0.118–0.415). By contrast, abstinence rates in the methadone-maintenance and alcoholic groups were, respectively, 0.177 (95% CI: 0.065–0.335) and 0.237 (95% CI: 0.098–0.409), as shown in Figure 2.

The alcohol disorder group obtained the best initial results (i.e. at weeks 1–12) but a high percentage of these patients relapsed during the first three months, especially after the pharmacological treatment was completed. Results in the other two groups were more stable over time (Figure 2). There were no differences between groups in adherence to pharmacological treatment.

Our team assessed the safety of varenicline in a previous study^22^. In the present study, we again evaluated safety, finding that varenicline was safe in this sample. The adverse events observed in the psychiatric disorder group in our sample were: dry mouth, flatulence, abnormal dreams, and nausea. According to diagnostic groups, the patients using methadone presented more adverse events than the alcohol group, but we did not observe other significant differences across diagnostic groups^22^. In our previous study, we reported that reducing the dose to 1 mg/day improved treatment adherence in patients who experienced adverse events and we also found that varenicline did not induce any significant exacerbations of psychiatric symptoms^22^.

## DISCUSSION

The aim of the present study was to determine the one-year outcomes of a multimodal treatment for smoking cessation intervention consisting of varenicline plus psychological treatment in a sample of alcoholics in remission, methadone-maintenance patients, and patients with psychotic disorders. Our results show that this multimodal therapy can help patients with addictive and psychiatric disorders to quit smoking. Although the success rates in these patient groups were somewhat lower in the first weeks than observed in smokers in the general population^11^, the results at weeks 24 and 52 were similar^16,25^.

Despite the relatively small sample size in this study, our results are consistent with those reported in studies with larger sample sizes. The EAGLES trial evaluated smoking cessation in a large sample (n=4074) of psychiatric patients, although patients with addictions were excluded in the EAGLES trial, continuous abstinence rates were 18.3% from weeks 9–24^11^. Grant et al.^26^ evaluated smoking cessation in alcoholic patients, reporting abstinence rates of 24% at week 4 and 21% at week 9; notably, their six months results (22%) were similar to our outcomes (22.5%). Hurt et al.^27^ recently published a study with a smaller sample than ours but with similar conclusions, varenicline is safe and efficacious for increasing smoking abstinence rates in smokers with alcohol abuse or dependence.

By contrast, Nahvi et al.^28^ evaluated 70 methadone-maintained patients treated with varenicline, reporting an abstinence rate at six months of only 8.6%; while the reason for this lower rate compared to ours is not clear; it may be at least partially due to the type of psychotherapy offered in multimodal approach given that Nahvi et al. administered the same pharmacological treatment.

Compared to the EAGLES trial, the patient sample in our study had more severe psychopathological disorders and a greater level of tobacco dependence. However, the samples in both studies achieved similar abstinence rates during the pharmacological treatment phase, which decreased sharply in both studies after finalization of pharmacological treatment. This finding supports the efficacy of varenicline in these patient populations, although it is worth noting that varenicline was somewhat less effective in these patients than in the general population.

Hitsman et al.^29^ concluded that the combination of cognitive behavioral therapy with motivational strategies and pharmacotherapy could improve smoking cessation outcomes in patients at mental health centers. Our findings confirm this conclusion, as we achieved good outcomes by using a comprehensive treatment approach (i.e. a multimodal intervention) at all participating centers. In our treatment program, all of the treating therapists were either psychologists or psychiatrists with extensive training in psychiatric pathology and addictions. In addition, the treatment centers were all reference centers for psychiatry, addictions, and smoking cessation. The participating centers were members of the working group on mental health and smoking, promoted by the Catalan Network of Smoke-Free Hospitals, and are thus especially prepared and motivated to provide smoking cessation aid to mental health patients.

One impediment to improving the outcomes of smoking cessation programs is the difficulty of standardizing non-pharmacological interventions. Numerous studies have evaluated pharmacological treatments, but few have assessed the different types of psychological interventions, and even fewer have investigated the influence of using a combined approach involving both pharmacological and psychological interventions. The present study adds valuable data, but additional observational or—ideally—randomized controlled studies are needed to better assess the factors described by Hitsman et al.^29^.

Few studies have evaluated tobacco cessation in methadone-maintained patients. However, the studies carried out to date in this population have reported six-month cessation rates of 5.2%^30^, 5.4%^31^ and 8.6%^28^; by contrast, we obtained a cessation rate of 17.7%. Perhaps our results are better due to the multimodal treatment, although it is difficult to make a comparison between studies when talking about psychological intervention, because many of them do not explain what type of intervention they perform.

Patients in the methadone group participate in a harm reduction program, but have not gone through the process of giving up the addictive substance and therefore would not benefit from the relapse prevention experience of alcoholic patients. Several studies suggest that the experience of giving up one drug is useful when trying to give up other drugs^26^. Continued drug use, even if the drug is methadone, may interfere with patients’ cognitive abilities and brain neurochemistry, making it harder for them to achieve and maintain abstinence^32^.

An important finding of our study is that, of all the patient-related baseline variables, the number of previous smoking cessation attempts was the strongest predictor of success, a finding that is consistent with previous reports^33^. However, the factors that trigger quit attempts in this population are poorly understood and more studies are needed to better characterize the factors that motivate multiple quit attempts. Motivation, as other authors reported^34^, or aspects related to skill acquisition, could be among the factors that facilitate quitting smoking and also prevent relapses. Whatever the relevant factor, it seems reasonable to point out that interventions that promote quit attempts may improve the likelihood of mid- to long-term smoking cessation.

### Study strengths and limitations

The main limitation of this study is the small sample size. Nevertheless, our findings were consistent with those obtained in studies involving larger sample sizes, such as the EAGLES trial and others^11,26^. Another potential limitation is the risk of variation in treatment due to the multicenter study design (11 different centers and a large number of therapists). Although the intervention was standardized by a common protocol, some therapist-related and location-related differences are possible. However, these differences are common in real-world clinical practice. In fact, a strength of this study is that it was conducted in a real clinical environment. Moreover, this study is one of the few to assess the efficacy of varenicline in an addicted population. Moreover, the one-year follow-up is longer than most other studies, which usually have a shorter follow-up period, lasting only until the end of pharmacological treatment. Moreover, we also measured exhaled CO levels to verify patient-reported abstinence. Finally, because the drug treatment was fully funded and closely monitored, we were able to closely assess treatment adherence, which is not always the case, due to the economic cost of the treatment, in a real-world environment such as a clinical setting that can confound results.

## CONCLUSIONS

The results obtained in this study support the use of multimodal treatment with varenicline in patients with alcohol addiction in remission, patients on methadone maintenance, and patients with stable psychotic disorders. Previous smoking cessation attempts were predictive of smoking cessation success in these patients. The high smoking prevalence rates in these patient populations impose a high burden in terms of morbidity and mortality. For this reason, it is essential to better identify the factors that would most likely improve the outcomes of smoking cessation treatments in these patient populations.

## CONFLICTS OF INTEREST

The authors declare that they have no competing interests, financial or otherwise, related to the current work. C. Pinet reports personal fees and non-financial support from Pfizer, outside the submitted work. The rest of the authors have also completed and submitted an ICMJE form for disclosure of potential conflicts of interest.
